# Characterization of Salt-Induced Epigenetic Segregation by Genome-Wide Loss of Heterozygosity and its Association with Salt Tolerance in Rice (*Oryza sativa* L.)

**DOI:** 10.3389/fpls.2017.00977

**Published:** 2017-06-08

**Authors:** Min Li, Wen-Sheng Wang, Yun-Long Pang, Jessica R. Domingo, Jauhar Ali, Jian-Long Xu, Bin-Ying Fu, Elec B. Venus, Zhi-Kang Li

**Affiliations:** ^1^School of Science, Anhui Agricultural UniversityHefei, China; ^2^Institute of Crop Sciences, Chinese Academy of Agricultural SciencesBeijing, China; ^3^International Rice Research InstituteLos Banos, Philippines; ^4^Shenzhen Institute for Breeding and Innovation, Chinese Academy of Agricultural SciencesShenzhen, China

**Keywords:** salt tolerance, rice, transgenerational epigenetic inheritance, designed QTL pyramiding, loss of heterozygosity

## Abstract

In a breeding effort to develop salt tolerant (ST) rice varieties by designed QTL pyramiding, large numbers of progenies derived from four crosses between salt- or drought- tolerant BC_2_F_5_ IR64 introgression lines, were subjected to severe salt stress, resulting in 422 ST plants. The progeny testing of the selected F_3_ lines under more severe salt stress resulted in identification of 16 promising homozygous lines with high levels of ST. Genetic characterization of the 422 ST F_3_ progeny and 318 random F_2_ plants from the same four crosses using 105 segregating SSR markers lead to three interesting discoveries: (1) salt stress can induce genome-wide epigenetic segregation (ES) characterized by complete loss of heterozygosity (LOH) and nearly complete loss of an allele (LOA) in the F_3_ progenies of four rice populations in a single generation; (2) ∼25% of the stress-induced ES loci were transgenerational and inherited from their salt- and drought- selected parents; and (3) the salt-induced LOH and LOA loci (regions) appeared to contain genes/alleles associated with ST and/or drought tolerance. 32 genomic regions that showed one or more types of salt-induced ES in the random and salt-selected progenies from these crosses. The same or different types of ES were detected with two large genomic regions on chromosomes 1 and 6 where more and the strongest ES were found across different populations. 14 genomic regions were found where the salt-induced ES regions were overlapping with QTL affecting ST related traits. The discovery of the three types of salt-induced ES showed several interesting characteristics and had important implications in evolution and future breeding for developing stress-resilient rice and crops.

## Introduction

Rice (*Oryza sativa* L.) is the most important staple food for half the world population. Grown in a wide range of diverse environments, rice crop encounters many abiotic stresses, of which salinity is the most important one affecting more than 20 million ha of rice lands along the coastal areas of tropical Asia and Africa (Li and Xu, 2007). Experimental evidence for environmentally induced transgenerational epigenetic inheritance was limited to a few well-characterized traits and loci in plants.

Loss of heterozygosity (LOH) and loss of an allele (LOA), frequently come hand in hand, are common phenomena attributed to some kinds of external selection in both natural and experimental populations of many diploid organisms (Rosenberg, 2011; Ford et al., 2015). For example, LOH was observed in large genomic regions of natural populations of diploid isolates of yeast (*Saccharomyces cerevisiae*) and interpreted as a result of selection from nutrient stress (Granek and Magwene, 2010). LOH was also observed in the vegetable pathogen *Phytophthora capsici* under chemical mutagenesis (Hulvey et al., 2010). Recently, by raising experimental populations derived from intra- and inter-specific hybrids of yeast in nutrient limited conditions for 100s of generations and sequencing the resulting cultures (Smukowski Heil et al., 2017) those were able to determine that LOH at a phosphate transporter gene, PHO84, result from selection for specific allele at this locus. In humans, LOH is commonly associated with cancerous cells (Knudson, 1985; Chigira et al., 1993). Recently, a strong correlation was found between LOH events at loci Mfd41, Tp53-Alu, and Mfd28 and the incidence of secondary tumors of breast cancer (Kamat et al., 2012). Similarly, LOH/LOA on specific chromosomal regions was reported to contribute to the glioma progression in Malay population (Zainuddin et al., 2004). In plants, the maize paramutations at loci *b1* and *r1* which encode two transcription factors for the pigment anthocyanin phenotypically behave like LOH/LOA since only one of the parental alleles at the loci is expressed in heterozygotes (Brink et al., 1996). In rice, greatly reduced heterozygosity was reported in the drought-, salt- and submergence-selected BC progenies (Li and Zhang, 2013; Wang et al., 2015), which actually resulted in a greatly accelerated breeding process to reach complete homozygosity by 3–5 years (Ali et al., 2017). Unfortunately, this type of stress-induced LOH in rice were not fully characterized and appropriately interpreted because of no inclusion of the random populations in these experiments. Many important questions remain unanswered regarding how many different types of epigenetic segregation (ES) can be induced by a specific environmental perturbation, how the environmentally induced epigenetic changes can pass across generations, and to what extent the environmentally induced epigenetic variation occur in a genome, etc.

Here we report, for the first time, a case of genome-wide salt (drought)-induced transgenerational and non-transgenerational ES characterized as LOH and LOA in several rice populations in breeding for developing salt tolerant (ST) rice varieties by designed QTL pyramiding. Our results provided compelling evidence for the genetic and epigenetic control of ST in rice.

## Materials and Methods

### Plant Materials

In a backcross breeding program initiated in 1998, IR64, the most widely grown rice variety in South/Southeast Asia, was used as recurrent parent to cross and backcross twice with six donors, Madhukar (India), Binam (Iran), OM1723 (Vietnam), FR13A (India), Hao-An-Nong (China), and BR24 (Bangladesh) (**Table 1**) to develop BC_2_F_2_ bulk populations as described previously (Lafitte et al., 2006). In 2001 dry season, the BC_2_F_2_ bulk populations were screened under severe terminal drought that killed most individuals of each BC population. A small portion of the BC progeny survived drought stress and was selected. The selected BC_2_F_3_ progeny were planted under the normal irrigated conditions for seed increase and genotyped with polymorphic SSR markers. The BC_2_F_4_ progeny were confirmed for improved drought tolerance (DT) in a replicated experiment (Lafitte et al., 2007) and were screened under EC (electric conductivity) 12 dSm^-1^ at the seedling stage in the 2002 wet-season. From these progeny, eight BC_2_F_5_ introgression lines (ILs) that showed significantly improved ST or DT and good yield performances under the normal conditions, were used as the parents to make four intercrosses (**Table 1**) to develop ST varieties by pyramiding ST QTLs from different donors. Two types of progeny were developed from each of the crosses, including a random set of F_2_ progeny obtained from F_1_ plants grown under the normal conditions and a set of ST F_3_ progeny selected under severe salt stress from 600 F_2_ individuals of each of the pyramiding F_2_ populations.

**Table 1:** The four crosses made between eight salt tolerant IR64 introgression lines (ILs) and their derived random F_2_ progeny and selected ST F_3_ progeny.

	Female parental ILs			Male parental ILs			Salinity selected			Random
				
Cross	Trait^a^	Line	Donor	Trait	Line	Donor	N1^b^	N2	SR (%)^c^	N3
A	ST	BSA25-1	Madhukar	ST	BSA36-1	Binam	600	143	23.3	80
B	DT	DGI245	OM1723	DT	DGI312	FR13A	600	65	10.8	72
C	DT	DGI388	Hao-An-Nong	DT	DGI94	BR24	600	76	12.7	85
D	DT	DGI381	Hao-An-Nong	DT	DGI300	FR13A	600	138	23.0	81
Total							2400	422	17.5	318


*^a^ST and DT indicate that the parental ILs were originally selected for salt and drought tolerance, respectively, in their original BC_*2*_ generations (Ali et al., 2006; Lafitte et al., 2006); ^b^N1, N2, and N3 are the F_*2*_ population size, the number of survived F_*2*_ plants (the F_*3*_ lines used in progeny testing), and number of random F_*2*_ plants from the non-stressed F_*1*_ plants of each cross (N2 and N3 plants were used for genotyping); and ^c^SR, survival rate.*

### Field Trials and Trait Measurements

#### Screening for Seedling Salt Tolerance (ST)

In the 2005 dry season, 600 F_2_ seeds from each cross were treated for 4 days at 44°C in a convection oven to break seed dormancy. The F_2_ seeds were then surface sterilized with fungicide and rinsed well with distilled water. Sterilized seeds were placed in petri dishes with moistened filter papers and incubated at 30°C for 48 h to germinate. Then, the germinated seeds were sown directly onto nylon mesh supported on floating Perspex grids. The grids were floated on large (1 m^2^) interconnected tanks (total volume 0.5–1.0 m^3^) at a rate of two pre-germinated seeds per hole on the floating Perspex grids (each grid contains 100 holes). The parental lines each was sown in five holes in a single row on each grid as the controls. The tanks were filled with distilled water. Water was replaced by a salinized nutrient solution at EC 6 dSm^-1^ at the two-leaf stage. Three days after initial salinity treatment, the salt concentration in the tanks was increased to EC 12 dSm^-1^ and maintained for 1 week. Then, the salt concentration of the solution was raised to EC 18 dSm^-1^ and maintained for 10 days. The salty solution in each tank was renewed every 5 days and maintained daily at pH of 5.0. The screening was conducted in the IRRI phytotron under the condition at 29/21°C day/night temperature and the minimum relative humidity of 70%. The standard score of visual symptoms of salt toxicity was recorded 16 days after initial salinization as described in the rice Standard Evaluation System (IRRI, 2013). Finally, when the two parents of each cross were completely killed, a total of 419 plants survived the stress, including 140, 65, 76, and 138 individuals from crosses (populations) A, B, C, and D, respectively. All survival plants were transferred to the field after being neutralized in the non-salt solution for 2 days. At the maturity, all seeds from the selected ST F_3_ plants were bulk harvested. In addition, a total of 318 random F_2_ plants were collected from the non-stressed F_1_ plants of each cross, including 80, 72, 85, and 81 plants for crosses A, B, C, and D, respectively (**Table 1**).

### Progeny Testing and Genotyping

The ST F_3_ progeny selected from the four populations were then progeny tested in the replicated experiments under salt stress during the dry season of 2006–2007. For each F_3_ line, 20 plants were evaluated with its parental ILs and IR64. The salt treatment was the same as the F_2_ screen described above, except after the salt concentration in the tanks was increased to EC 18 dSm^-1^ and maintained for 5 days, and then the salt concentration was further raised to EC 24 dSm^-1^ and maintained until IR64 and the parental ILs were all dead. Then, the survival plants of each F_3_ lines were counted. Leaf tissues from 20 plants of each of the F_3_ lines from each cross was bulk-harvested for DNA extraction and genotyping to reconstruct the genotypes of their original F_2_ plants. A total of 637 well-distributed anchor SSR markers^1^ from the Cornell University were used to survey the polymorphic markers differentiating the parental ILs of each cross, from which 35, 29, 6, and 25 differentiating SSR markers were used to genotype the selected ST F_3_ progeny and the random F_2_ plants from each cross.

## Results and Discussion

### Segregation in the Random and/or Salt Stressed Progeny

#### Selection Efficiency of ST from Different Pyramiding Populations

**FIGURE 1:**
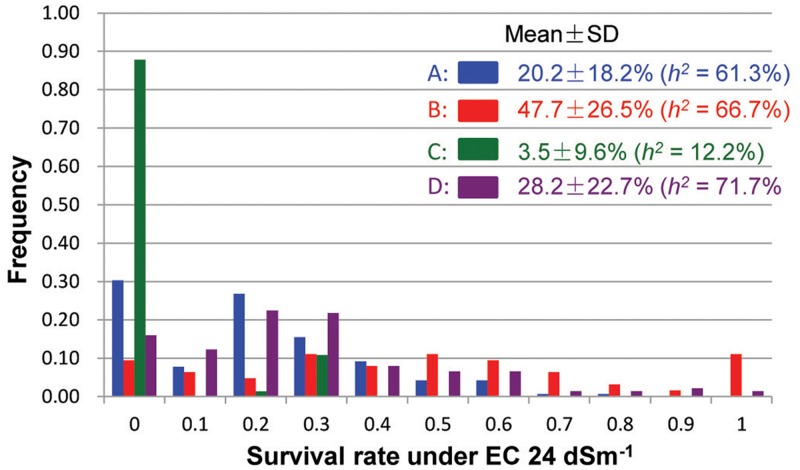
Frequency distributions of survival rates (under EC 24 dSm^-1^) of the salt-selected F_3_ progeny from four F_2_ populations derived from intercrosses between two introgression lines of rice.

Under the severe salinity of the 2007 wet-season, 422 plants of the pyramiding F_2_ populations survived and produced seeds. The survival rate differed considerably among different populations, with populations A and D showing ∼23% of survival rates, and populations B and C exhibiting 11–13% of survival rates (SR) (**Table 1**). In the F_3_ progeny testing, the realized heritability, *h^2^* (the percentage of F_3_ progeny showing significant improved ST, or transgressive segregation) was fairly high for populations A (61.3%), B (66.7%) and D (71.7%), but very low for population C (12.2%). However, progeny from population B showed the highest level of ST with a mean survival rate of 47.7 ± 26.5%, followed by populations D and A (28.2 ± 22.7% and 20.2 ± 18.2%). Again, progeny from population C showed lowest survival rate at 3.5 ± 9.6%. Most importantly, of the 422 selected progeny, 11 F_3_ lines from cross B and 7 lines from cross D exhibited high levels of ST with survival rates ≥ 80% under EC 24 dSm^-1^ (**Figure 1**).

### Salt-Induced Epigenetic Segregation Characterized by Loss of Heterozygosity (LOH) and Loss of Allele (LOA)

**FIGURE 2:**
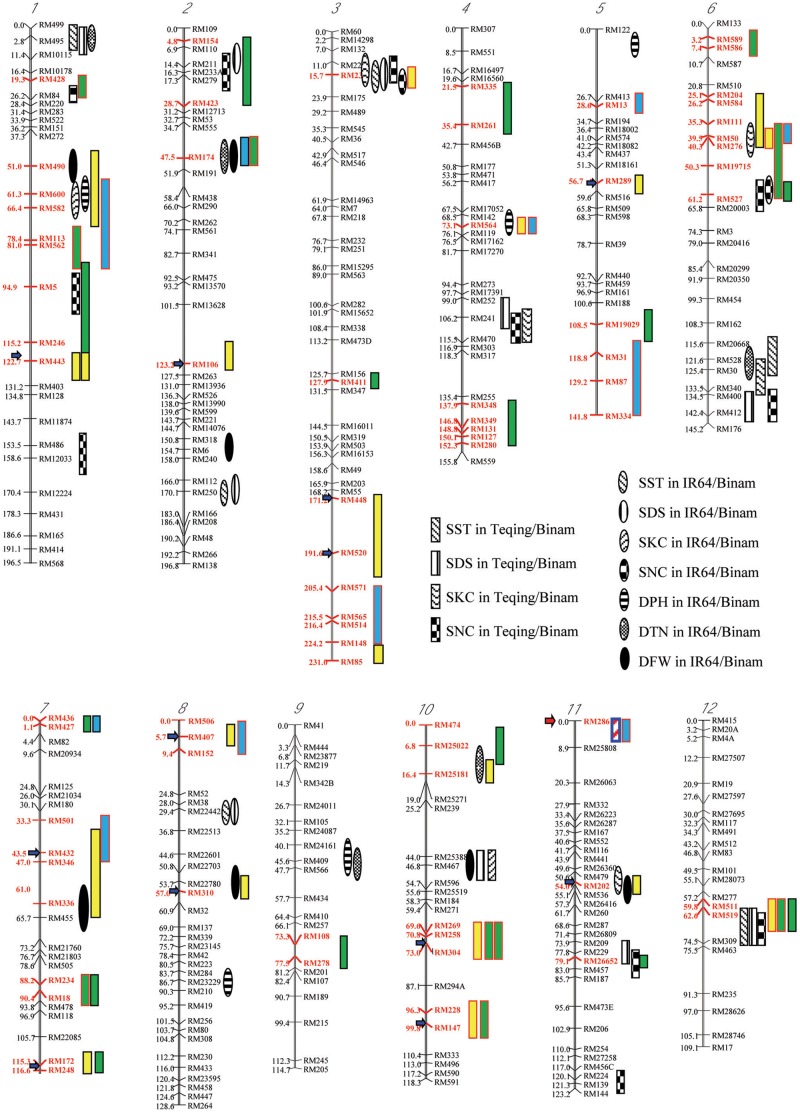
Chromosomal distributions of regions (red markers) showing two types of salt-induced epigenetic segregation (loss of heterozygosity) detected in four F_2_-F_3_ populations derived from intercrosses between drought selected introgression lines, and their associations with QTLs affecting six traits for salt tolerance (Zang et al., 2008), in which the red and black outlined boxes indicate that the favorable alleles are from the maternal and paternal introgression line parents, respectively; while the boxes filled with yellow, green, and blue indicated the types I, II, and III of epigenetic segregation, respectively. The salt tolerance related traits, SST, SDS, SKC, SNC, DPH, DTN, and DFN present.

**Table 2** and **Figure 2** show the segregation patterns of all segregating SSR markers in the 422 ST F_3_ lines and 318 random F_2_ progeny from the four populations, which revealed three types of extremely distorted or ES characterized with loss of heterozygosity (LOH) and loss of allele (LOA, fixation of one of the parental alleles) in the random and/or salt-selected progeny. Of the 20 segregating markers (regions) examined in the progeny from cross A, 13 markers on 9 chromosomes (1–3, 5–8, 10, and 11) were of type I, which had a mean heterozygosity (H) of 1.9 and 0.4% in the random and salt-selected progenies, respectively (**Table 2A**). The paternal alleles almost reached fixation with a mean frequency of 0.931 (0.958) at 11 of the 12 loci in the random (salt-selected) progeny, except for RM171 on chromosome 10 at which the maternal allele was almost fixed with an allele frequency of 0.913 (0.993), indicating those alleles of ES were inherited from their parents. The remaining seven loci on seven chromosomes (2, 4–7, 10, and 11) were of type II that showed the 1:2:1 Mendelian segregation ratio in the random F_2_ progeny but strong ES in the salt-selected progeny with a mean H of 12.8%. The paternal alleles at five of these type II ES loci were favored with a mean frequency of 0.717, while the maternal alleles at the remaining two loci were favored with a mean frequency of 0.649.

**Table 2A:** Genomic regions (20 SSR markers) showing epigenetic segregation in the random and/or salt stressed progeny derived from cross A between two salt tolerant IR64 ILs.

				Random population (N_A_ = 80)					Salt-selected population (N_A_ = 143)				
					
Cross	Type	Marker	Bin	F_(A)_	F_(B)_	F_(H)_	*X^2^*_(1A:2H:1B)_	*P*-value	F_(A)_	F_(B)_	F_(H)_	*X^2^*_(1A:2H:1B)_	*P*-value
A	I	RM600	1.3	0	1	0	240	7.70E-53	0.015	0.985	0	389.2	3.00E-85
A	I	RM443	1.8	0	1	0	240	7.70E-53	0	1	0	420	6.30E-92
A	I	RM106	2.6	0.013	0.988	0	232.1	4.00E-51	0.083	0.917	0	318.3	7.70E-70
A	I	RM520	3.1	0.075	0.725	0.2	96.4	1.20E-21	0	1	0	417	2.80E-91
A	I	RM85	3.12	0	1	0	234	1.50E-51	0.036	0.964	0	381.4	1.50E-83
A	I	RM289	5.3	0	0.975	0.025	221.3	8.80E-49	0.014	0.971	0.014	388.6	4.10E-85
A	I	RM584	6.3	0.038	0.963	0	216.9	8.00E-48	0.036	0.964	0	381.4	1.50E-83
A	I	RM432	7.5	0.013	0.987	0	229.1	1.80E-50	0.036	0.964	0	381.4	1.50E-83
A	I	RM172	7.8	0.026	0.974	0	215.4	1.70E-47	0.071	0.929	0	183.8	1.20E-40
A	I	RM301	8.2	0.012	0.988	0	232.1	4.00E-51	0.036	0.964	0	206	1.90E-45
A	I	RM25181	10.2	0.013	0.987	0	226.1	8.00E-50	0.021	0.979	0	396.5	7.90E-87
A	I	RM171	10.5	0.913	0.088	0	188.9	9.60E-42	0.993	0.007	0	412.1	3.30E-90
A	I	RM202	11.3	0.076	0.924	0	192.6	1.50E-42	0.04	0.96	0	339.6	1.80E-74
A	II	RM154	2.1	0.325	0.138	0.538	6.1	4.80E-02	0.029	0.971	0	388.9	3.50E-85
A	II	RM335	4.1	0.278	0.342	0.38	5.2	7.40E-02	0.063	0.598	0.339	75.9	3.40E-17
A	II	RM19029	5.7	0.247	0.247	0.506	0	9.90E-01	0.029	0.971	0	388.9	3.50E-85
A	II	RM589	6.1	0.247	0.195	0.558	1.5	4.80E-01	0.619	0.075	0.306	99.7	2.20E-22
A	II	RM234	7.7	0.342	0.184	0.474	4	1.40E-01	0.479	0.371	0.15	71.8	2.50E-16
A	II	RM474	10.1	0.308	0.244	0.449	1.5	4.80E-01	0	0.993	0.007	412	3.40E-90
A	II	RM26652	11.3	0.218	0.295	0.487	1	6.10E-01	0.101	0.804	0.094	227.3	4.50E-50


*A, B, and H represent homozygous genotypes of the female, male parents and the heterozygotes.*

Of the 20 segregating markers examined in the progenies of cross B, three loci in chromosomes 1, 4, and 6 were of type I ES, which showed extreme LOH in both the random (*H* = 1.5%) and salt-selected (*H* = 1.8%) progenies significant at *P* < 1.2^-17^. However, both parental alleles had significant frequencies ≥ 25% in the random F_2_ progeny, while one of the parental alleles became virtually fixed at all three loci in the salt-selected progeny (**Table 2B**). We detected 12 loci of type II ES on eight chromosomes (1–4, 6, 7, 9, 10, and 12) in cross B that showed the expected 1:2:1 segregation in the random F_2_ progeny but extreme ES in the salt-selected progeny. The paternal alleles at 9 of the 12 loci were fixed or nearly so with frequencies > 0.943, and so were for the maternal alleles at the remaining three loci in the salt-selected progeny. There were three type III loci that showed ES in both the random and salt-selected progenies, but their favorable alleles were switched in the random and selected progenies. We noted two loci near RM174 and RM519 on chromosomes 4 and 12, at which the random progeny did not show LOH, but had significantly higher frequencies of the maternal alleles. In the salt-selected progeny, the two loci showed extreme ES. Thus, these two loci should be considered type II ES loci.

**Table 2B:** Genomic regions (20 SSR markers) showing epigenetic segregation in the random and/or salt stressed F_2_ progeny derived from cross C between two drought tolerant IR64 ILs.

				Random population (N_B_ = 72)					Salt-selected population (N_B_ = 65)				
					
Cross	Type	Marker	Bin	A	B	H	*X^2^*_(1A:2H:1B)_	*P*-value	A	B	H	*X^2^*_(1A:2H:1B)_	*P*-value
B	I	RM443	1.8	0.25	0.75	0	108	3.50E-24	0	1	0	177	3.70E-39
B	I	RM564	4.5	0.676	0.279	0.044	78	1.20E-17	0.947	0	0.053	147.9	7.50E-33
B	I	RM276	6.3	0.639	0.361	0	83.1	9.00E-19	1	0	0	156	1.30E-34
B	II	RM428	1.1	0.324	0.225	0.451	2.1	3.60E-01	0.982	0.018	0	157.1	7.50E-35
B	II	RM246	1.8	0.239	0.211	0.549	0.8	6.70E-01	0	0.962	0.038	140.5	3.20E-31
B	II	RM423	2.2	0.264	0.222	0.514	0.3	8.60E-01	0	1	0	177	3.70E-39
B	II	RM174	2.3	0.403	0.181	0.417	9.1	1.10E-02	0.091	0.909	0	128.6	1.20E-28
B	II	RM411	3.8	0.167	0.361	0.472	5.7	5.90E-02	0.017	0.966	0.017	161.4	9.10E-36
B	II	RM261	4.3	0.239	0.282	0.479	0.4	8.30E-01	0	1	0	159	3.00E-35
B	II	RM127	4.8	0.264	0.292	0.444	1	6.10E-01	0	1	0	174	1.60E-38
B	II	RM527	6.4	0.3	0.243	0.457	1	6.20E-01	0.966	0.034	0	158.6	3.70E-35
B	II	RM427	7.1	0.236	0.25	0.514	0.1	9.60E-01	0	0.982	0.018	163.1	3.80E-36
B	II	RM248	7.8	0.292	0.194	0.514	1.4	4.90E-01	0.057	0.943	0	136.4	2.50E-30
B	II	RM278	9.7	0.222	0.208	0.569	1.4	4.90E-01	0	1	0	177	3.70E-39
B	II	RM304	10.5	0.328	0.284	0.388	3.6	1.60E-01	1	0	0	168	3.30E-37
B	II	RM511	12.4	0.125	0.236	0.639	7.3	2.60E-02	0	0.983	0.017	166.1	8.50E-37
B	II	RM519	12.4	0.507	0.101	0.391	26	2.30E-06	1	0	0	171	7.40E-38
B	III	RM436	7.1	0.736	0.222	0.042	98.5	4.00E-22	0	1	0	168	3.30E-37
B	III	RM501	7.3	0.456	0.544	0	69.1	1.00E-15	1	0	0	177	3.70E-39
B	III	RM334	5.9	0.324	0.507	0.169	35.9	1.60E-08	0.549	0.275	0.176	29	4.90E-07


*A, B, and H represent homozygous genotypes of the female, male parents and the heterozygotes.*

In cross C, only five loci in three regions on chromosomes 1, 6, and 7 were segregating. All five loci were of type II ES that showed the Mendelian segregation in the random F_2_ progeny but extreme ES in the salt-selected progeny (**Table 2C**). The paternal alleles at four loci were predominant, while the maternal allele at the remaining locus was favored in the selected progeny. There were 14 loci segregating in cross D. Of these, only one locus on chromosome 3 was of type I ES that exhibited ES segregation in both the random and salt-selected progeny, though ES in the selected progeny was much more extreme with the maternal allele reaching the fixation (**Table 2C**). We detected four loci of type II ES in cross D that showed the Mendelian segregation in the random progeny but extreme ES in the salt-selected progeny. Surprisingly, there were nine loci of type III ES detected in cross D, at which extreme ES with complete LOH and LOA (fixation of the paternal alleles) at all loci except for one on chromosome 6 in the random progeny, but complete LOH and LOA (fixation of the maternal alleles) at all loci in the salt-selected progeny. In fact, all 138 salt-selected progeny in cross D were fixed at the maternal alleles from a *Geng* (*japonica*) donor, Hao-An-Nong at all 14 segregating loci.

**Table 2C:** Genomic regions (19 SSR markers) showing epigenetic segregation in the random and/or salt stressed progeny from populations derived from crosses C and D between two pairs of drought tolerant IR64 ILs.

				Random population (N_C_ = 85, N_D_ = 81)					Salt-selected population (N_C_ = 76, N_D_ = 138)				
					
Cross	Type	Marker	Bin	A	B	H	*X^2^*_(1A:2H:1B)_	*P*-value	A	B	H	*X^2^*_(1A:2H:1B)_	*P*-value
C	II	RM113	1.5	0.212	0.247	0.541	0.8	6.70E-01	1	0	0	228	3.10E-50
C	II	RM5	1.7	0.259	0.235	0.506	0.1	9.50E-01	0.132	0.868	0	158.5	3.80E-35
C	II	RM246	1.8	0.271	0.153	0.576	4.3	1.10E-01	0.013	0.987	0	220.1	1.60E-48
C	II	RM527	6.4	0.188	0.235	0.576	2.4	3.10E-01	0.053	0.947	0	197.7	1.20E-43
C	II	RM18	7.7	0.259	0.247	0.494	0	9.80E-01	0.171	0.829	0	141.8	1.60E-31
D	I	RM231	3.2	0.726	0.274	0	102.8	4.70E-23	1	0	0	414	1.30E-90
D	II	RM174	2.3	0.222	0.272	0.506	0.4	8.20E-01	1	0	0	414	1.30E-90
D	II	RM402	6.3	0.272	0.272	0.457	0.6	7.40E-01	1	0	0	414	1.30E-90
D	II	RM258	10.5	0.275	0.2	0.525	1.1	5.80E-01	1	0	0	414	1.30E-90
D	II	RM519	12.4	0.259	0.309	0.432	1.9	3.90E-01	1	0	0	414	1.30E-90
D	III	RM600	1.3	0	1	0	243	1.70E-53	1	0	0	414	1.30E-90
D	III	RM514	3.12	0	1	0	243	1.70E-53	1	0	0	414	1.30E-90
D	III	RM564	4.5	0	1	0	243	1.70E-53	1	0	0	414	1.30E-90
D	III	RM13	5.2	0	1	0	243	1.70E-53	1	0	0	414	1.30E-90
D	III	RM31	5.9	0	1	0	243	1.70E-53	1	0	0	414	1.30E-90
D	III	RM87	5.8	0	1	0	243	1.70E-53	1	0	0	414	1.30E-90
D	III	RM50	6.3	0.272	0.728	0	114.8	1.20E-25	1	0	0	414	1.30E-90
D	III	RM407	8.1	0	1	0	243	1.70E-53	1	0	0	414	1.30E-90
D	III	RM286	11.1	0	1	0	243	1.70E-53	1	0	0	414	1.30E-90


*A, B, and H represent homozygous genotypes of the female, male parents and the heterozygotes.*

### Characteristics of Genomic Regions Showing Salt-Induced ES and Their Associations with ST QTL

Taking the above results together, we detected a total of 32 genomic regions that showed one or more types of salt-induced ES in the random and salt-selected progenies from the four pyramiding crosses (**Figure 2**). When results from different crosses were compared, we noted several interesting characteristics of the salt-induced ES. First, the size of genomic regions covered by multiple closely linked markers showing salt-induced ES detected in specific crosses varied considerably, ranging from a single marker to a group of closely linked markers covering a region > 30 cM. Second, the same type of ES or different types of ES were detected in progenies from different crosses with two large genomic regions on chromosomes 1 and 6 where more and the strongest ES were detected across different populations. Thirdly, in our previous study, we reported 27 genomic regions that harbor 53 QTLs affecting six ST related traits identified in the random backcross progenies of two crosses, IR64/Binam and Teqing/Binam with Binam as the donor (Sun et al., 2007; Zang et al., 2008), which are highly related to the populations used in this study. These ST related traits included scores of salt toxicity of leaves (SST), survival days of seedlings (SDS), shoot N^+^ concentration (SNC), shoot K^+^ concentration (SKC), plant height difference of the stress to control (DPH), tiller number difference of the stress to control (DTN), and plant fresh weight difference of the stress to control (DFW). When taking a close look at their genomic locations, we found 14 genomic regions where the salt-induced ES regions were overlapping with QTL affecting ST related traits. This is much higher than the probability (*P* ≤ 0.03) caused by chance, suggesting that ST QTL regions tended to show ES in the salt-selected progeny.

In this study, we have shown the results from our breeding effort to develop ST rice varieties by the approach of “designed QTL pyramiding (DQP)” (Li and Xu, 2007; Li and Zhang, 2013). The development of 18 completely homozygous ST lines that showed > 80% survival rates under the natural whole-growth duration salt stress of EC 12–18 dSm^-1^ in 3 years has demonstrated the power and efficiency of this breeding strategy. During this breeding process, we also sought to understand the genetic basis of ST in rice. The discovery of the three types of salt-induced ES was surprising, each of which showed several interesting characteristics and had important implications in evolution and future breeding for developing stress-resilient rice and crops.

### Characteristics of Salt (Drought)-Induced ES

In this study, although a small number of markers were segregating in each population, the fact that all of them showed strong LOH and/or LOA in the salt-selected progenies of all four populations, indicating that salt induced ES occurred across the whole genome. Of the genomic regions showing ES, 14 (25%) of them were type I ES which showed LOH and/or LOA in both the random and salt-selected progenies of three crosses (**Figure 2**), 12 of which were detected in cross A whose parental ILs were all salt-selected (Ali et al., 2006). The remaining four regions detected in crosses B and D whose parents were all drought selected (Lafitte et al., 2006). Clearly, these type I ES loci were inherited from their parents. In other words, this type of ES loci showed transgenerational inheritance. In rice, reduced heterozygosity was reported in the drought-, salt- and submergence-selected BC progenies (Li and Zhang, 2013; Wang et al., 2015; Ali et al., 2017), but this type of stress-induced LOH were not fully characterized and appropriately interpreted. Thus, the inclusion of the random populations in this experiment provided compelling evidence that type I ES loci were inherited from their parental ILs. We noted that in almost all cases, the levels of ES (LOH and LOA) in the salt-selected progenies were stronger than in the random progenies from the same crosses, further implicating the important role of salt stress for the observed LOH and LOA in the populations. We noted that the behaviors of type III ES loci were very similar to type I ES loci except that the observed LOA in the random progeny was in the opposite direction as that in the salt-selected progeny from the same cross. When taking a close look at the 12 (9 in cross D and 3 in cross B) loci of type III ES identified, we found that 2 (RM501 on bin 7.3 and RM334 on bin 5.9) of them in cross B were not typical (**Table 2**). In the former case, LOH was observed and the frequencies of the parental alleles did not differ significantly in the random progeny, but showed LOH and LOA in the salt-selected one. Thus, these loci should be considered as a type I ES locus. In the latter case, there was equally reduced heterozygosity and no LOH or LOA observed in the random and salt-selected progenies. The remaining 10 loci (9 from cross D and 1 from cross B) were of typical type III ES or transgenerational ES, at which LOH and LOA were present in both the random and salt-selected progenies, but different parental alleles were fixed in the random and salt-selected progenies, respectively. As mentioned earlier, the parental ILs of crosses B and D were all drought-selected. ST and DT are known to be partially overlapped genetically and physiologically (Zhou et al., 2009; Hong et al., 2016). As expected, no type III ES loci were identified in cross A whose parental lines were all salt-selected. Moreover, there were apparent correspondences between the salt-induced ES loci and QTL affecting ST traits identified in the random ILs of the same IR64 backgrounds (**Figure 2**). All these results suggested that type I ES regions contain the parental alleles contributing to both ST and DT, while different parental alleles in type III ES regions were associated separately with ST and DT, respectively. If so, our results would suggest that the ES regions indeed contain genes for ST and/or DT as the targets of the strong phenotypic selection for ST and/or DT in our breeding processes.

Different from types I and III ES loci, the 21 type II ES loci from the four crosses showed the Mendelian 1:2:1 segregation in the random progenies but LOH/LOA in the salt-selected progenies of the same crosses, provided compelling evidence that the observed ES at all these loci were salt-induced during the selection process. Interestingly, the overall levels of LOH, particularly LOA, in the type II ES regions were less pronounced as compared to those of types I and III (**Table 2**), suggesting that the effect of the applied abiotic stress (salt and/or drought) on the level of LOH and LOA was accumulative. It should be pointed out that the genotypes of all selected survival F_2_ plants were reconstructed from the bulk DNA of their F_3_ progeny. Thus, we were actually measuring the gametic genotypes of the selected F_2_ plants. Thus, the observed genome-wide ES resulted from strong salt (drought)-induced gametic selection favoring one of the parental alleles in the salt-stressed F_2_ plants of all crosses.

Environmentally induced genome-wide LOH and/or LOA have been reported in both natural and experimental populations of many diploid organisms, such as in natural and experimental populations of diploid isolates of yeast as a result of selection on nutrient stress (Granek and Magwene, 2010; Smukowski Heil et al., 2017). In humans, a strong correlation was found between LOH events and deficient protein expression of specific genes and the incidence of secondary tumors of breast cancer (Kamat et al., 2012). LOH/LOA on specific chromosomal regions was reported to contribute to the molecular pathway of glioma progression in Malay population (Zainuddin et al., 2004). The second one is that LOH and/or LOA in the genome of an organism can be a localized event involving specific genes in specific genomic region, or genome-wide events. Then, questions arise regarding what molecular mechanism(s) were responsible for the observed stress-induced ES in rice and other organisms.

### Stress-Induced Genomic Imprinting

We noticed that ‘stress-induced genomic imprinting (SIGI)’ or ‘stress imprinting’ (Zucchi et al., 2012), an epigenetic phenomenon, was the most likely molecular mechanism responsible for the salt-induced LOH and LOA in rice observed in this study based on two indirect pieces of evidence. First, the SIGI is a well-characterized epigenetic molecular mechanism that leads to differential expression of maternal and paternal alleles, depending on their parent-of-origin (Feil and Berger, 2007). In fact, the SIGI behaves remarkably similar to the salt (drought)-induced epiloci of this study in the following ways. First, like the type II ES loci, environmentally induced epigenetic changes are transient in most cases (Iwasaki, 2015), and in some cases they are stably maintained through mitotic cell divisions and show transgenerational inheritance, as demonstrated in vernalization of Arabidopsis by prolonged cold that involves epigenetic silencing of specific genes (Kim and Sung, 2012; Song et al., 2012). In Arabidopsis, SIGI results from stress regulated expression and function of three key enzymes, MET1, DDM1, and MOM1, which regulate, by DNA methylation, the expression of some Arabidopsis loci under heat stress and set or reset stress-induced chromatin changes and epigenetic marks transmitted to the next generation (Tittel-Elmer et al., 2010). Secondly, in a sister line of the parental ILs (crosses B and D) selected from drought, we observed that drought was able to induced site-specific DNA methylation/demethylation across the genome (Wang et al., 2010), and ∼25% of those drought-induced DNA methylation/demethylation sites were irreversible, while the 75% were reversible when the drought stress was removed. This lent a strong support to the above speculation that drought- or salt-induced DNA methylation was the most likely molecular mechanism for the observed salt- or drought induced ES, because the proportions of those irreversible and reversible methylated sites matched almost perfectly with that of type I (III) and II ES, even though it remains a mystery how this happened at the molecular level.

## Conclusion

The observed genome-wide salt- and drought-induced LOH and LOA are good news for plant breeders because strong selection under salt and/or drought could fix large number of segregating targeting loci in 1–2 generations.

## Author Contributions

Z-KL and J-LX designed the experiment; W-SW, Y-LP, JD, and EV performed all the phenotypic evaluation; ML and W-SW performed analysis and interpretation of the data; ML, W-SW, and Y-LP drafted the manuscript; Z-KL, W-SW, JA, and B-YF revised the MS; all authors revised the paper and approved the final version to be published.

## Conflict of Interest Statement

The authors declare that the research was conducted in the absence of any commercial or financial relationships that could be construed as a potential conflict of interest.

## Footnotes

1 http://www.gramene.org/
